# In vitro and in vivo gene transfer in the cloudy catshark *Scyliorhinus torazame*

**DOI:** 10.1111/dgd.12824

**Published:** 2022-11-30

**Authors:** Chika Fujimori, Chie Umatani, Misaki Chimura, Shigeho Ijiri, Hisanori Bando, Susumu Hyodo, Shinji Kanda

**Affiliations:** ^1^ Atmosphere and Ocean Research Institute The University of Tokyo Kashiwa Chiba Japan; ^2^ Department of Biological Sciences, Graduate School of Science The University of Tokyo Bunkyo‐ku Tokyo Japan; ^3^ Graduate School of Fisheries Sciences Hokkaido University Hakodate Hokkaido Japan; ^4^ Division of Applied Bioscience, Research Faculty of Agriculture Hokkaido University Sapporo Hokkaido Japan; ^5^ Present address: Optics and Imaging Facility National Institute for Basic Biology Okazaki Aichi Japan; ^6^ Present address: Division of Applied Biological Chemistry, Graduate School of Agriculture Tokyo University of Agriculture and Technology Fuchu‐shi Tokyo Japan

**Keywords:** baculovirus, cartilaginous fish, elasmobranch, electroporation, gene transfer

## Abstract

Cartilaginous fishes have various unique physiological features such as a cartilaginous skeleton and a urea‐based osmoregulation strategy for adaptation to their marine environment. Also, because they are a sister group of bony vertebrates, understanding their unique features is important from an evolutionary perspective. However, genetic engineering based on gene functions as well as cellular behavior has not been effectively utilized in cartilaginous fishes. This is partly because their reproductive strategy involves internal fertilization, which results in difficulty in microinjection into fertilized eggs at the early developmental stage. Here, to identify efficient gene transfer methods in cartilaginous fishes, we examined the effects of various methods both in vitro and in vivo using the cloudy catshark, a candidate model cartilaginous fish species. In all methods, green fluorescent protein (GFP) expression was used to evaluate exogenous gene transfer. First, we examined gene transfer into primary cultured cells from cloudy catshark embryos by lipofection, polyethylenimine (PEI) transfection, adenovirus infection, baculovirus infection, and electroporation. Among the methods tested, lipofection, electroporation, and baculovirus infection enabled the successful transfer of exogenous genes into primary cultured cells. We then attempted in vivo transfection into cloudy catshark embryos by electroporation and baculovirus infection. Although baculovirus‐injected groups did not show GFP fluorescence, electroporation successfully introduced GFP into muscle cells. Furthermore, we succeeded in GFP transfer into adult tissues by electroporation. The in vitro and in vivo gene transfer methods that worked in this study may open ways for genetic manipulation including knockout experiments and cellular lineage analysis in cartilaginous fishes.

## Graphical abstract

We attempted in vitro and in vivo gene transfection into cloudy catshark by various gene transfer methods. We successfully introduced exogenous genes by means of lipofection, electroporation and baculovirus infection in vitro. Furthermore, in vivo electroporation effectively introduced exogenous genes into cloudy catshark embryos and adult tissues.

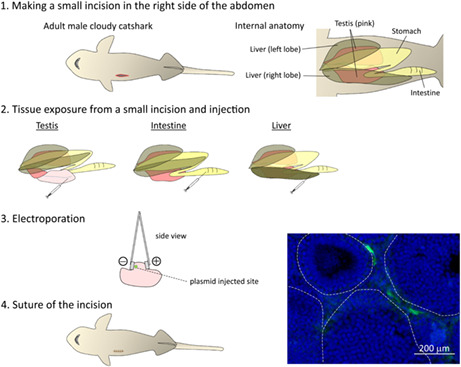

## 1 INTRODUCTION

Cartilaginous fishes have various unique biological characteristics such as a cartilaginous skeleton and a urea‐based osmoregulation strategy for adaptation to their marine environment. They also show diverse reproductive strategies, including oviparity and placental viviparity (Hamlett, 2005; Hyodo et al., 2014; Smith, 1936). These fishes are an extant sister group of bony vertebrates, which means that a thorough understanding of their biology will yield important information on the evolution of vertebrate physiology (Hara et al., 2018; Inoue et al., 2010; Venkatesh et al., 2014). Despite the importance of cartilaginous fish research from this and other perspectives, our understanding of the physiology of cartilaginous fish remains relatively rudimentary. This may be due to several characteristics of cartilaginous fish that make them difficult to study, including their long life cycle, their largely pelagic habitat, and their large size.

Because these characteristics are minimized in the cloudy catshark (*Scyliorhinus torazame*), it is a promising candidate model cartilaginous fish species. First, they are rather small and easy to maintain in a laboratory aquarium. Second, they are oviparous and spawn approximately once every 2–3 weeks in laboratory tanks (Inoue et al., 2022), so developing embryos are easily obtained. Furthermore, genomic data acquired through whole‐genome sequencing are available, which makes various molecular biological analyses easier (Hara et al., 2018; Kuraku, 2021).

Even in this model species, however, the limitations in experimental approaches, especially difficulty in genetic manipulation, remain a serious issue. The main factor limiting genetic manipulation of these species is their reproductive strategy: all cartilaginous fish species reported to date are copulating animals (Carrier, 2004; Hamlett, 2005). Even in oviparous species, the fertilized egg is encapsulated in an egg case and retained in the oviduct for a certain period before egg laying occurs. This reproductive feature makes it difficult to perform microinjection into fertilized eggs at the early developmental stages. There are several reports of gene transfer into germ cells in vertebrates, e.g., gene transfer into sperm nuclei in *Xenopus laevis* (Kroll & Amaya, 1996), spermatogonia in mouse and zebrafish (Huang et al., 2000; Kurita et al., 2004), and primordial germ cells in birds (Motono et al., 2010), which may be another way to generate transgenic animals in species with internal fertilization. For both analyses using transient forced expression and generation of transgenic animals, we considered it an important first step to establish an efficient method of introducing exogenous genes into in vivo tissues. Thus far, however, there has been only one report of gene transfer into cartilaginous fish cells in vitro, in which the researchers attempted lipofection and electroporation in a cell line derived from dogfish shark embryos (Parton et al., 2007). Furthermore, there is only one report of in vivo gene transfer in cartilaginous fish using little skate embryos (Jung et al., 2018). Thus, both in vitro and in vivo gene transfer are still challenging in cartilaginous fishes.

For exogenous gene transfer into vertebrate cells, three methods are commonly used: physical methods such as electroporation; chemical methods such as lipofection or the use of cationic polymers or polyethylenimine (PEI); and biological methods using viruses. Gene transfer using viruses has been reported in a limited number of species (Huang et al., 2011; Kawasaki et al., 2009; Overturf et al., 2003) and has never been attempted in cartilaginous fishes. In the present study, to identify efficient methods of gene transfer in cloudy catsharks, we first attempted gene transfer methods in vitro and in vivo.

## 2 MATERIALS AND METHODS

### 2.1 Animals

Captive cloudy catsharks (*Scyliorhinus torazame*) and their naturally spawned eggs were transported from Ibaraki Prefectural Oarai Aquarium to the Atmosphere and Ocean Research Institute at the University of Tokyo. They were reared in a 1000‐L tank with recirculating natural seawater at 16°C under a constant photoperiod of 12 h light/12 h dark. The embryos were staged according to previous studies (Ballard et al., 1993; Honda et al., 2020). All experiments were conducted in accordance with the Guidelines for Care and Use of Animals approved by the ethics committee of the University of Tokyo (P19‐2).

### 2.2 Baculovirus preparation

A bacmid containing inverted repeats of the *piggyBac* transposon, CMVp‐AcGFP, and SV40p‐Hyg (a hygromycin resistance gene) was constructed through modification of a pFastBac1‐Δpolh plasmid (Yokoo et al., 2013). These sequences were obtained from pPIGA3GFP (inverted repeats of the *piggyBac* transposon; Tamura et al., 2000) and the pAcGFP‐Hyg C1 vector (CMVp‐AcGFP and SV40p‐Hyg) (632,492; Takara Bio USA). Recombinant baculovirus was then produced using a Bac‐to‐Bac system and Sf9 cells. The baculovirus titer was measured by GFP expression in HEK293A cells and P2 supernatant with 1.55 × 10^5^ gene transfer units (gtu)/ml was obtained.

### 2.3 In vivo transfection of cloudy catshark embryos and adult tissues

**FIGURE 1:**
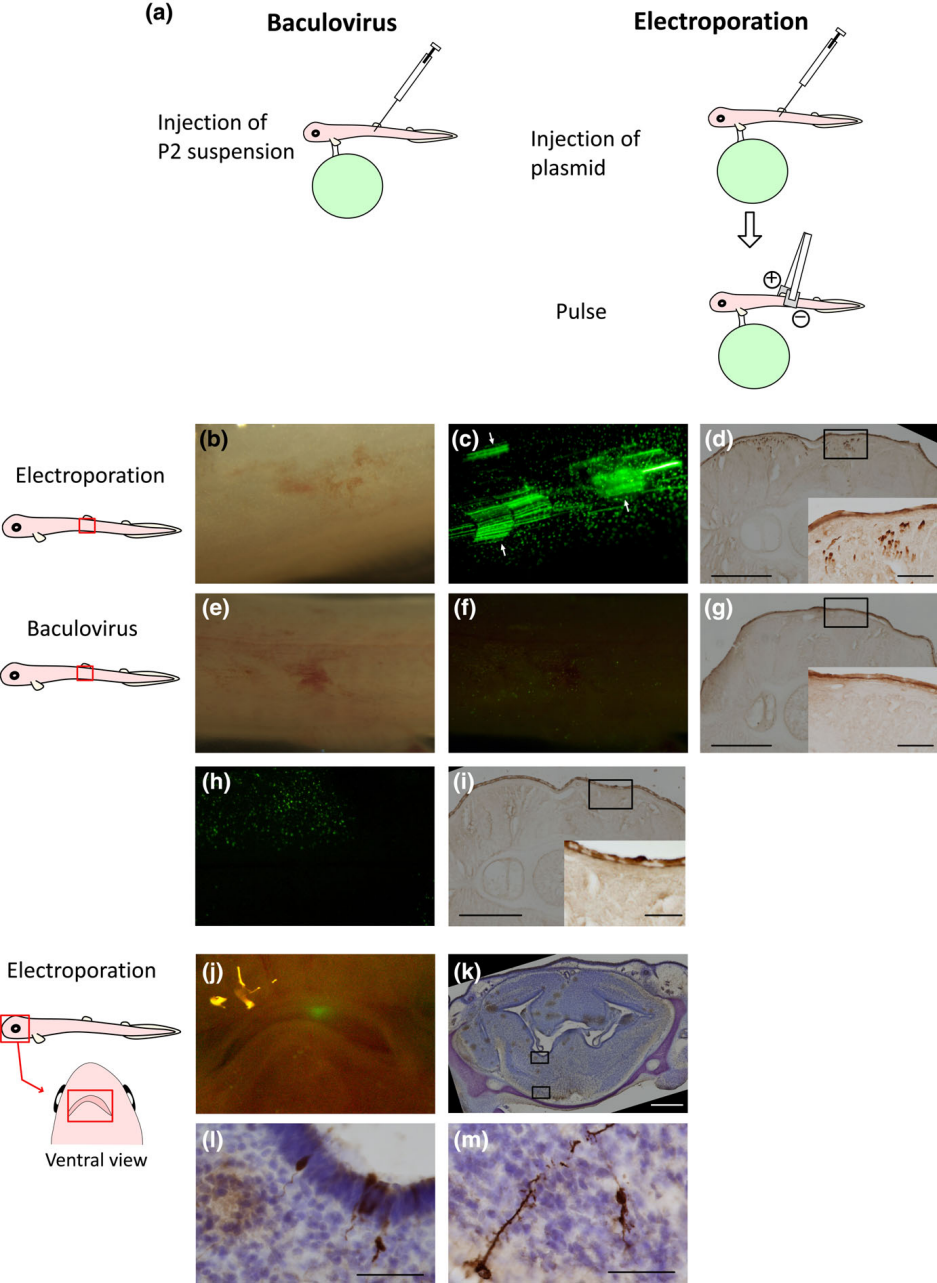
In vivo gene transfer into cloudy catshark embryos by electroporation or baculovirus infection. (a) Procedures for baculovirus infection and electroporation. In baculovirus infection (left panel), P2 supernatant was injected. In electroporation (right panel), after injection of the plasmid, electric pulses were applied at the site of injection. In both baculovirus infection and electroporation, injection was performed at the lateral body under the first dorsal fin. (b–i) Results of gene transfer into cloudy catshark embryos. Bright field (b, e) and fluorescence images (c, f) of cloudy catshark embryos 7 days after electroporation (b, c, *n* = 3) or baculovirus infection (e, f, *n* = 5). GFP fluorescence was clearly observed in the cylinder‐shaped muscle of the injected area of an embryo subjected to electroporation (c, arrows), but not in the specimen injected with baculovirus (f) or in untreated embryos (h). Note that dot fluorescence observed in (c, f, h) represents autofluorescence of the skin. (d, g, i) Immunohistochemistry for EGFP of the transverse sections of embryos also showed that EGFP‐immunoreactive cells were only observed in the plasmid‐injected side of the electroporated embryos and not in baculovirus‐infected embryos (g) and the serial section of the contralateral side of the plasmid‐injected side of electroporated embryos (i). Note that the upper side of the figure represents the lateral side, the left side represents the ventral side, and the right side represents the dorsal side of the body. (j) Catshark embryos whose brains had been subjected to electroporation. GFP fluorescence was observed at the injected site. (k) EGFP immunohistochemistry using transverse sections of (j) indicated that EGFP‐immunoreactive neurons were localized in the subpallium. (l, m) Magnified images of the boxed regions of (k). Scale bars represent 500 μm (d, g, i, k) and 50 μm (insets of d, g, i, l, m).

**FIGURE 2:**
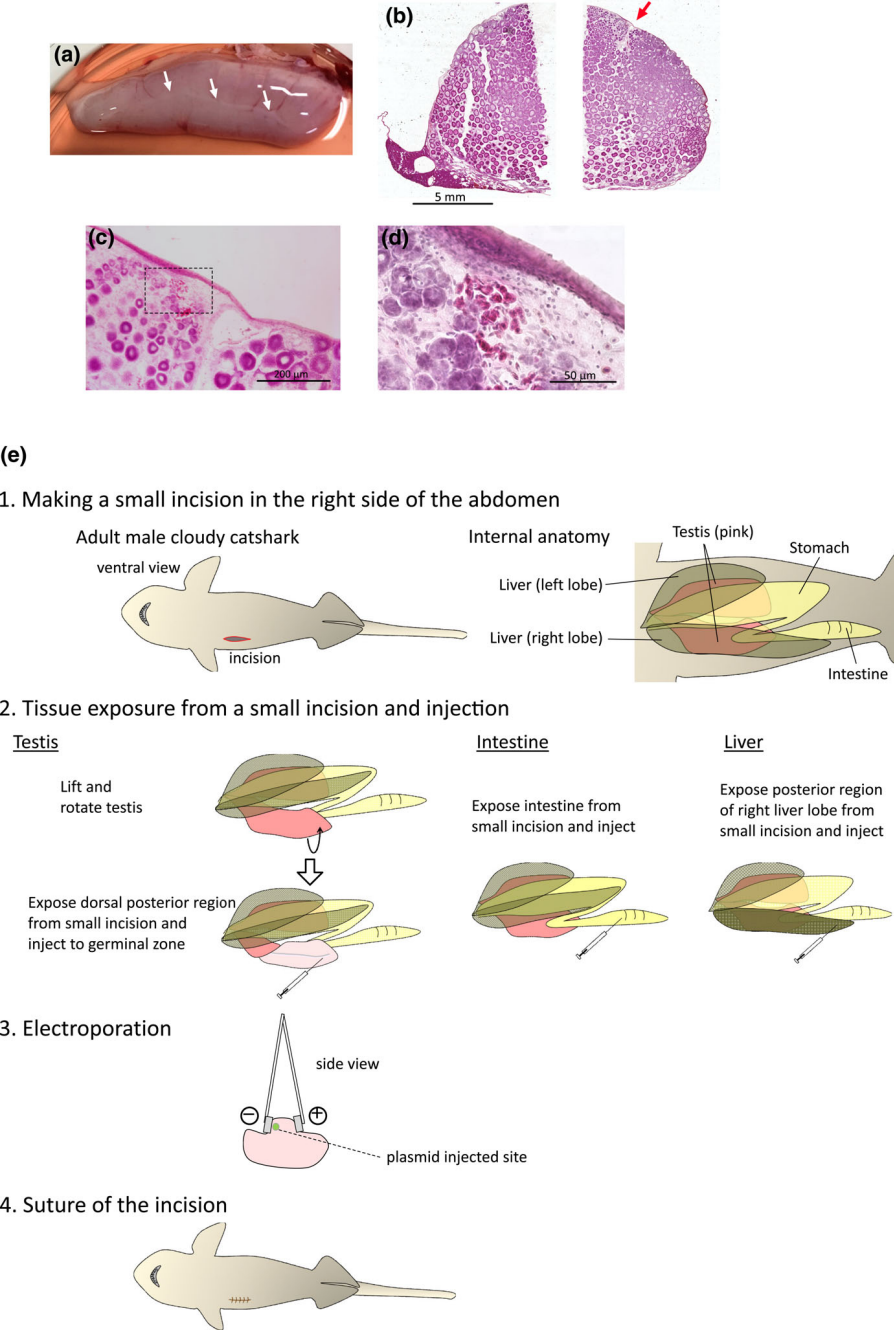
Morphology of the adult cloudy catshark testis and the procedure of electroporation of adult tissues. (a) Dorsal side of a dissected adult cloudy catshark testis. White arrows show the cord‐like structure, which is the germinal zone where plasmid was injected prior to electroporation. (b) Hematoxylin–eosin staining of transverse sections of adult cloudy catshark testes. Because of the large size of the cloudy catshark testis, the testis was cut into two parts, medial (left) and lateral (right), and cryosections were prepared. The arrow indicates the germinal zone, and the spermatocysts were differentiated radially from this area. (c) Magnified image of the germinal zone. Connective tissue near the germinal zone was observed in the section, and it corresponds to a white cord‐like structure on the surface of the whole mount testis. (d) Magnified image of the boxed area of (c). (e) Procedure of electroporation into adult cloudy catshark tissues. The cloudy catshark abdominal cavity, liver, digestive tract including stomach and intestine, and testis are located from the ventral side. For electroporation into adult tissues, the tissues were exposed through a small incision in the right side of the abdomen. Following plasmid injection, electric pulses were applied with the negative electrode on the injected side. After electroporation, we sutured the incision. Scale bars represent 5 mm (b), 200 μm (c), and 50 μm (d).

**FIGURE 3:**
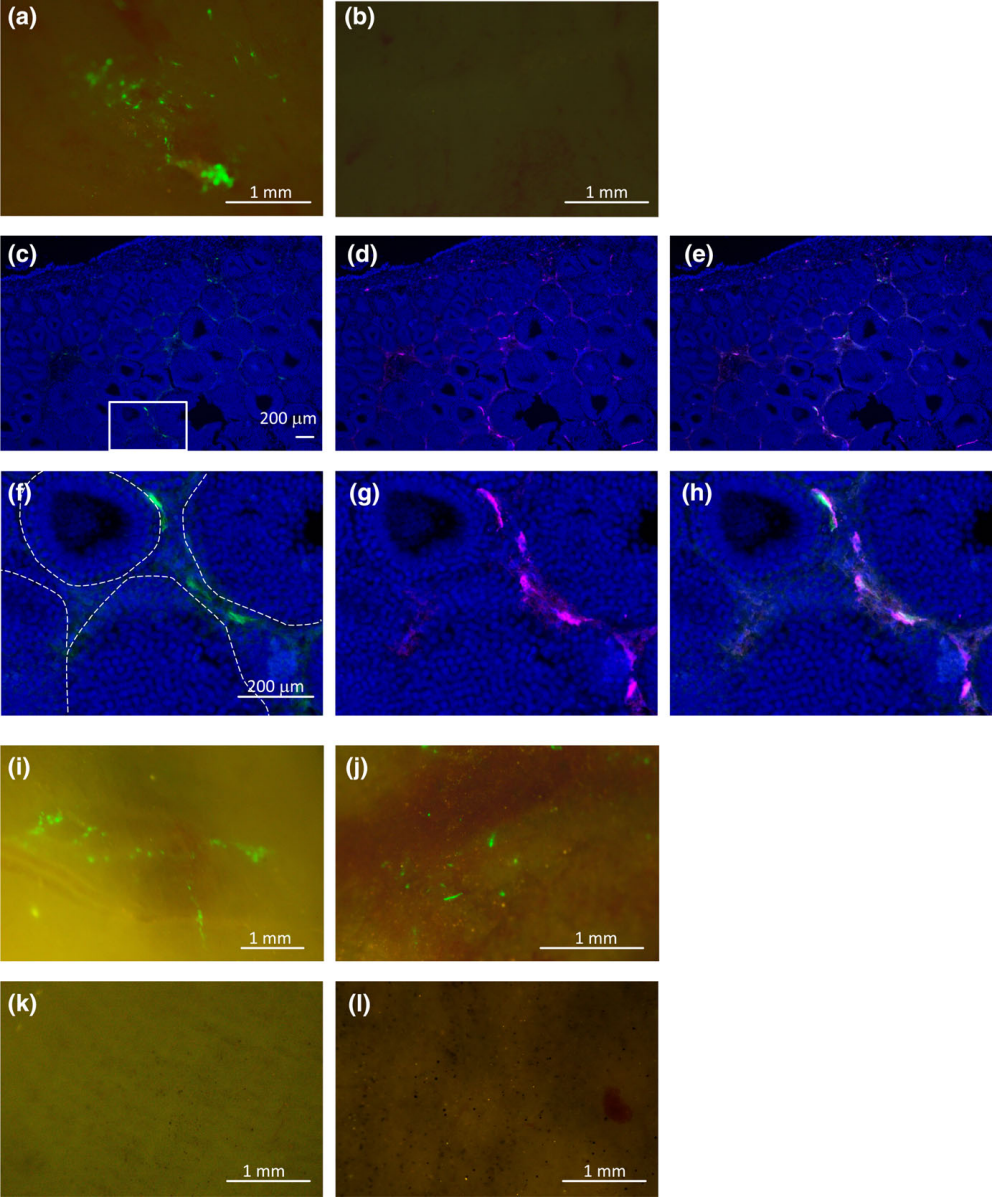
In vivo gene transfer into adult cloudy catshark tissues. Fluorescence images of an adult cloudy catshark testis 7 days after electroporation (a) and an untreated testis (b). (c–h) EGFP signals in the testis section. Raw GFP fluorescence (c, f), which is confirmed to show GFP immunoreactivity (d, g: GFP‐immunoreactive signal [magenta], e, h: merge) outside of spermatocysts. (f), (g), and (h) are magnified images of the boxed areas of (c), (d), and (e), respectively. Note that the germinal zone was localized away from this picture where GFP was observed. Dashed lines indicate the spermatocysts. Fluorescence images of the adult cloudy catshark intestine and liver 7 days after electroporation are shown in (i) and (j). (k, l) Fluorescence images of untreated intestine (k) and liver (l). Scale bars represent 1 mm (a, b, i–l) and 200 μm (c–h).

Embryos removed from egg capsules at stage 31L to 32 were anesthetized with 0.02% ethyl 3‐aminobenzoate methanesulfonate (MS‐222) (Sigma‐Aldrich) in natural seawater, and baculovirus infection or electroporation was performed. Next, 1–2 μl of P2 supernatant (for baculovirus infection) or 0.5–2 μg of pEGFP‐N1 vector (Takara Bio USA) (for electroporation) was subcutaneously injected into the lateral body under the first dorsal fin (Figure 1a) using a 10‐μl Hamilton syringe #80300 (Hamilton Company). For electroporation, embryos were temporarily transferred to PBS (140 mM NaCl, 2.7 mM KCl, 10 mM PO_4_^3−^, pH 7.4). Embryo bodies were placed between handmade tweezer‐type electrode set with 10 × 8 mm square aluminum plates with the negative electrode on the injected side (Figure 1a). Electroporation was carried out using a T820 electroporator (BTX) at 15–50 V for 50 ms with two to eight square‐wave pulses. After transfection, all embryos were reared in sterilized seawater at 16°C with aeration for 7 days. The seawater for incubation was refreshed every 2 days. Seven days after gene transfer, we examined the embryos for GFP fluorescence. For in vivo electroporation into adult tissues, adult male catsharks (267.5–454.3 g) were anesthetized with 0.02% MS‐222 in natural seawater, and one of the testes was exposed from a small incision (~5 cm) in the abdomen (Figure 2e‐1,e‐2). Then, 50–100 μg of pEGFP‐N1 vector was injected into the liver, intestine, and testis (Figure 2e‐2), and five 50‐ms electric pulses were applied at 30 V with the electrode used for embryo electroporation such that the negative electrode was on the injected side (Figure 2e‐3). After electroporation, the incision was closed by suturing both the peritoneum and the epithelium (Figure 2e‐4), and the animals were reared in 16°C seawater with aeration. After 5–7 days, the cloudy catsharks were anesthetized and sacrificed. Their electroporated tissues were sampled, and GFP fluorescence was observed.

### 2.4 Histology

Five to seven days after in vivo transfection, cloudy catshark embryos or adults were anesthetized with 0.02% MS‐222. The injected areas were dissected and fixed with 4% paraformaldehyde in PBS. After cryosectioning as a previous study (Umatani et al., 2022), immunohistochemistry was performed according to a slightly modified version of the protocol described by Takahashi et al. (2016). Briefly, 25 (embryos) or 30 (adults) μm thick transverse cryosections were rinsed with PBS containing 0.5% triton X‐100 (PBST) and incubated with anti‐GFP rat monoclonal antibody (1:1000, GF090R; Nacalai Tesque Inc.) overnight at room temperature. After endogenous peroxidase activity was inactivated by the addition of 0.3% H_2_O_2_ in PBS for 30 min at room temperature, a secondary antibody reaction was performed using biotinylated anti‐rat IgG antibodies (1:200; Jackson ImmunoResearch). After washing with PBS, sections were incubated with VECTASTAIN® Elite ABC‐HRP kit reagent (Vector Laboratories, Inc.). GFP‐expressing cells were visualized with 3,3'‐diaminobenzidine (DAB) and 0.003% H_2_O_2_ or Alexa Fluor® 555 Streptavidin (Thermo Fisher Scientific). Embryonic brain sections were counterstained with cresyl violet acetate after DAB staining. For histological analysis of adult testes, transverse sections were prepared and stained with hematoxylin and eosin as previously described (Honda et al., 2020). The stained sections were photographed with an All‐in‐One Fluorescence Microscope BZ‐X810 (KEYENCE) with its automatic image stitching function.

## 3 RESULTS AND DISCUSSION

### 3.1 Lipofection, baculovirus infection, and electroporation were effective for gene transfer into cloudy catshark cells

We first tried in vitro gene transfer into cultured cells (see the Supplementary Text containing SUPPLEMENTARY MATERIALS AND METHODS and SUPPLEMENTARY RESULTS AND DISCUSSION in Appendix S1 and the Supplementary Materials containing Figures S1, S2, and S3 for more details). Briefly, we used embryos at stage 29–31E for the primary cell culture to avoid pre‐hatching, which occurs at the end of stage 31E (Honda et al., 2020) and may increase the risk of microbial contamination due to the influx of seawater to the inside of the egg capsule. Of the various gene transfer methods examined in this study, lipofection (3.6%, Figure S1), baculovirus infection (1.5%, Figure S1), and electroporation (0.59%, Figure S1) effectively introduced exogenous genes into cloudy catshark primary cultured cells (Table S1), whereas gene transfer was not successful in non‐treated controls (0.01%, Figure S1) and after PEI transfection (0.005%; Figure S1) or adenovirus infection (0%, Figure S1). The variation in transfection efficiency values within the same experimental conditions may be due to cell seeding density and embryonic cell heterogeneity. Although baculovirus was originally discovered as a virus that infects insect cells, successful gene delivery by baculovirus vectors has been reported in mammalian cell lines such as HEK cells and teleost cell lines or live embryos such as zebrafish (Huang et al., 2011; Kost & Condreay, 2002; Wagle & Jesuthasan, 2003). Here, we demonstrated that baculovirus can infect cloudy catshark primary cultured cells incubated at a low temperature with high osmotic pressure and urea concentration. This suggests that baculovirus can infect a wide range of hosts.

Electroporation and lipofection have been demonstrated to be effective not only in mammalian cell lines but also in teleost, dogfish shark, and invertebrate cell lines (Kobayashi & Belloncik, 1993; Parton et al., 2007; Vallone et al., 2007; Yuan, 2007). The present study shows that these methods can also be applied to cartilaginous fish primary cultured cells (Figure S1), which are suggested to better retain the physiological properties of the original cells when compared to established cell lines.

### 3.2 In vivo electroporation effectively introduced exogenous genes into cloudy catshark embryos and adult tissues

Followed by our methodological validation using primary cultured cells, we attempted in vivo gene transfer into embryos at stage 31L–32 through baculovirus infection and electroporation (Figure 1a). We chose these stages for in vivo transfection because they are sufficiently grown to exhibit many physiological processes such as nutritional absorption through the digestive tract and buccal pumping as well as a functional immune system, yet their skin is still not too thick (Honda et al., 2020; Lloyd‐Evans, 1993; Tomita et al., 2014). At 7 days after electroporation, GFP fluorescence was clearly observed in the skeletal muscles of the injected areas (Figure 1b,c); this expression was confirmed by EGFP immunohistochemistry of the transverse sections, indicating that the EGFP‐expressing cells were restricted to the injected areas (Figure 1d). Under the conditions of 0.5–2 μg plasmid injection and eight or less 50‐ms pulses at 30 V, fluorescence was observed in all survivors (Figure S4). In spite of its effective transfection into cultured cells in vitro, baculovirus failed to introduce a detectable level of GFP at injected sites (Figure 1e–g), similar to the uninjected embryos (Figure 1h). The reason for this discrepancy remains to be determined, but the differences between in vitro and in vivo situations, such as the immune system and intercellular adhesion in vivo, might play a role. No GFP‐positive muscle cell was observed in the section of the contralateral side of the electroporated embryo (Figure 1i).

In addition to muscle cells, EGFP was also detected in neuron‐like cells in the embryos in which we performed electroporation in the brain (Figure 1j–m). Previously, dyes such as DiI have been used as tracers in lineage tracing analyses in cartilaginous fish (Kuroda et al., 2021; Martin et al., 2016), which made it difficult to label specific cell types. In combination with the use of gene‐specific promoters, electroporation enables cellular lineage tracing of specific cells during development.

Finally, we attempted gene transfer into germ cells of adult testis and other adult tissues (Figure 2). Prior to electroporation, transverse sections of the cloudy catshark testis were prepared and the structure was observed. As reported for other related species (Callard et al., 1989; Hamlett, 2005; Pratt, 1988), germ cells form a spherical unit called a spermatocyst, in which germ cells synchronously undergo spermatogenesis (Figure 2b, Figure S5). The germinal zone, the area of the testis in which the undifferentiated germ cells are concentrated, was localized on the dorsal side of the testis (Figure 2b arrow,c,d), and connective tissue near the germinal zone corresponds to a white cord‐like structure on the surface of the whole mount testis (Figure 2a white arrows). When meiosis proceeds, chromatin remodeling and subsequent chromosome packaging occur in testicular germ cells (Martinage et al., 1985). Since integration of transgenes into the host genome is supposed to be difficult at this stage, we attempted gene transfer into more undifferentiated germ cells, namely spermatogonia, in this study. Thus, we injected plasmid into this white cord‐like structure of the adult testis and performed electroporation in vivo (Figure 2e). One week after electroporation, GFP fluorescence was observed in the testis (Figure 3a), while GFP fluorescence was not detected in untreated testes (Figure 3b). Here, we observed some EGFP‐positive cells in the regions away from the germinal zone (Figure 3c–e), suggesting that they have migrated from the germinal zone along with germ cell differentiation (Figure 2b). On the other hand, we could not find positive germ cells (inside the spermatocysts) in the histological observation of testes (Figure 3c–h). One possible reason for this is the presence of a basement membrane lining the spermatocyst structure (Callard et al., 1989; Hamlett, 2005) preventing the plasmid to penetrate into the spermatocysts. In other cartilaginous fish such as spiny dogfish shark (*Squalus acanthias*) and bonnethead shark (*Sphyrna tiburo*), it has been reported that more undifferentiated germ cells (primary spermatogonia) do not have a cyst structure (Betka & Callard, 1999; Hamlett, 2005; Parsons & Grier, 1992). If such undifferentiated germ cells are identified by in situ hybridization to label germ cell‐specific genes such as *vasa* and *nanos* in cloudy catshark testes in the future, gene transfer into germ cells by electroporation and the generation of genetically modified offspring from resulting sperm will become more feasible. Also, we succeeded in gene transfer in a part of the intestine and liver (Figure 3i–l), which suggests this method can be applied to various tissues. Based on this transfer method of DNA fragments, functional analysis by gene overexpression and/or knockdown in the cells of adult tissues can be performed in the future.

### 3.3 Possible future applications of gene transfer methods in cartilaginous fishes

In the present study, we successfully introduced exogenous genes by means of lipofection, electroporation, and baculovirus infection in vitro. These achievements will allow us to apply these gene transfer methods in various future studies investigating the development and physiology of cartilaginous fishes at the cellular level. For example, previous functional studies of the transporters or channels involved in osmotic regulation have been performed using *Xenopus* oocytes (Hasegawa et al., 2016). However, the methods described here will enable us to analyze the activities of transporters and channels in more nearly physiological conditions. Although the efficiency of these transfection methods is still low, single‐cell analysis methods such as patch clamp and Ca^2+^/cAMP imaging can be applied.

We also succeeded in in vivo gene transfer in both embryos and adults of cartilaginous fishes by means of electroporation, which allows in vivo forced expression locally at the site of electroporation. If an all‐in‐one CRISPR/Cas9 vector (Chu et al., 2015) is used for this application, gene knockdown in target cells is also possible. Thus, in the future, the transfer of a Cas9‐gRNA integrated vector may permit the generation of cloudy catsharks with local knockout of a specific gene by the CRISPR/Cas9 system. These techniques will likely use a reverse‐genetics approach in cartilaginous fishes. Future improvement of transfection efficiency and the development of a means of transfection of germ cell lines will make generation of transgenic cloudy catsharks more realistic.

## AUTHOR CONTRIBUTIONS

CF, SK, and SH designed the research. CF, CU, MC, and SK performed experiments. SI and HB contributed to the generation of baculovirus. SK and SH supervised the projects. CF prepared the draft of the manuscript and SK, CU, SI, and SH modified the manuscript. All authors read and approved the final manuscript.

## Supporting information


**Appendix S1.** Supporting information.

**Figure S1.** Gene transfer into primary cultured cells of cloudy catshark by various methods.
**Figure S2.** Efficiency of baculovirus‐mediated GFP transfection at various doses in vitro.
**Figure S3.** Efficiency of gene transfer under various electroporation conditions in cloudy catshark cells in vitro.
**Figure S4.** Results of electroporation under various conditions in cloudy catshark embryos in vivo.
**Figure S5.** Spermatocyst morphology of the adult cloudy catshark testis.
**Table S1.** The proportions of fluorescent cells after transfection of GFP‐containing vector or virus by various methods.
**Table S2.** The proportions of fluorescent cells after infection with different volumes of baculovirus. Statistical significance of differences from the control (cells treated with 0 L baculovirus) was assessed by Dunnett's multiple comparison test (*p < .05, **p < .01).
**Table S3.** The proportions of fluorescent cells after electroporation under various conditions. Statistical significance of differences from the control (cells subjected to electroporation at 0 kV) was assessed by Dunnett's multiple comparison test (*p < .05, **p < .01).
