# Supplementary material for: In vitro and in vivo gene transfer in the cloudy catshark Scyliorhinus torazame
Source: Dev Growth Differ. 2022 Nov 30;64(9):558–65. doi: 10.1111/dgd.12824 (PMC10099843; doi:10.1111/dgd.12824)
Supplement: Supplementary file 1 — Appendix S1. Supporting information. [file DGD-64-558-s002.pdf]

Supplementary Text

1. SUPPLEMENTARY MATERIALS AND METHODS

1.1 Primary cell culture

Embryos at stage 29-31E (before the pre-hatching period) were anesthetized with 0.02% ethyl 3-aminobenzoate methanesulfonate (MS-222) (Sigma-Aldrich, St. Louis, MO) in natural seawater and sacrificed by decapitation. After they had been cut into small pieces with sterilized scissors, embryonic tissues were incubated in a dissociation medium [L-15 medium (FUJIFILM Wako Pure Chemical Corporation, Osaka, Japan) containing 500 U/ml collagenase (FUJIFILM Wako Pure Chemical Corporation), 300 mM urea and 212.5 mM NaCl] for 1 hour at 16 °C with pipetting every 15 minutes. Cell suspension was washed with culture medium and spread onto a 24-well culture plate coated with 0.1% gelatin. The culture medium containing L-15 supplemented with 20 mM HEPES (pH 7.4), 300 mM urea, 180 mM NaCl, 20% FBS, 100 U/ml penicillin and 100 μg/ml streptomycin was replaced every three to four days. The osmolality of the dissociation medium and the culture medium was measured with a 5600 vapor pressure osmometer (Wescor, Logan, UT) and adjusted to ~1000 mOsm with NaCl.

1.2 Gene transfer to primary cultured cells

Lipofectamine and PEI transfection

Two days after primary cell culture was started, 0.8 μg of pEGFP-N1 vector (Takara Bio USA, Mountain View, CA), which carries EGFP under the promoter of human *Cytomegalovirus* (CMV), was transfected using 2 μl of Lipofectamine® 2000 (Thermo Fisher Scientific, Waltham, MA) or 2.5 μl of PEI-max (1.0 mg/ml) (Polysciences, Warrington, PA). Opti-MEM (Thermo Fisher Scientific) supplemented with 300 mM urea and 225 mM NaCl (~1000 mOsm) was used as the medium for transfection. After seven days of transfection, cells were examined for GFP fluorescence.

Adenovirus infection

Recombinant adenovirus for the expression of EGFP was constructed according to the method described by Toyoda *et al.* (Toyoda et al., 2016) with minor modifications. In brief, CRE-nls-GFP ORF was amplified from addgene plasmid #49056 and inserted into the adenoviral expression vector pAd/CMV/V5-DEST^{TM} Gateway Vector plasmid (Thermo Fisher Scientific). After production and amplification of the adenovirus, the virus was purified using an Adenovirus (Ad5) Purification and Concentration Kit (AdenoPACK 20; Sartorius, Goettingen, Germany) according to the manufacturer's instructions and stored at -80 °C until use. The titer of adenovirus was examined using an Adeno-XTM Rapid Titer Kit (Takara Bio USA) and determined to be 3.4×10^{11} ifu/ml. Two days after seeding the primary culture, 1 μl/well of the adenovirus was infected into the cells. Seven days after the transfection, cells were examined for GFP fluorescence.

Baculovirus infection

In the experiment validating this transfection method (Fig. S1), 5 μl of P2 supernatant was treated. To examine the infection efficiency of baculovirus (Fig. S2), 2-20 μl of P2 supernatants were added to cloudy catshark primary cultured cells two days after the culture was started. We examined the cells for GFP fluorescence seven days after infection.

Electroporation

Electroporation was performed immediately after cell dissociation from embryonic body. A dissociated cell suspension containing 50 ng/μl of pEGFP-N1 vector was inserted into 1-mm gap cuvettes and chilled on ice for 10 minutes. Electroporation was then conducted under various conditions using a T820 electroporator (BTX, Holliston, MA). The cell suspension was then chilled on ice for another 10 minutes and seeded onto 0.1% gelatin-coated dishes. In the experiment validating this transfection method (Fig. S1), electroporation was performed at 0.2 kV for 50 μsec with three pulses. We examined the cells for GFP fluorescence nine days after electroporation.

1.3 Examination of transfection efficiency in cell culture

Transfection efficiency was examined on day nine days after the start of cell culture, when GFP

fluorescence was observed sufficiently because of accumulation of expressed GFP in the transfected cells. Hoechst 33342 (NucBlue™ Live ReadyProbes™ Reagent, Thermo Fisher Scientific) was added according to the manufacturer's instructions. Then, 21.4 mm^2 of the contents of each well was photographed through an All-in-One Fluorescence Microscope BZ-X810 (KEYENCE, Osaka, Japan) equipped with the following filter set: Hoechst, ex. 360 ± 20/ em. 460 ± 25; GFP (EGFP, AcGFP), ex. 470 ± 20/ em. 525 ± 25. The numbers of total cells and fluorescence-positive cells within each captured area were counted using ImageJ. Note that a very small percentage of cells showed autofluorescence. Although it has different excitation/emission spectrum from the those of GFP, we could not distinguish between GFP and autofluorescence because the microscope used in this study was equipped with a band-pass filter for fluorescence observation. However, this inevitable occurrence of pseudo-positive cells shown in control samples was extremely lower than GFP. Therefore, we statistically analyzed the effect of each gene transfer method by comparing to the non-transfected control.

1.4 Statistical analysis

All values are shown as mean ± standard deviation (SD). The transfection efficiencies of baculovirus infection and electroporation in cloudy catshark primary cell culture were analyzed using Dunnett's multiple comparison test, and the significance of the difference from control conditions was assessed. p-values less than 0.05 were considered statistically significant.

2. SUPPLEMENTARY RESULTS AND DISCUSSION

2.1 Gene transfer into cloudy catshark cells

In cloudy catshark primary culture cells, lipofection (3.6%, Fig. S1b), baculovirus infection (1.5%, Fig. S1e) and electroporation (0.59%, Fig. S1f) effectively introduced exogenous genes. Especially, in baculovirus infection, gene transfer efficiency was depended on treatment volume at least the range we examined (Table S2, Fig. S2). In electroporation, the gene transfer efficiencies were higher at 0.2 kV for 50 μsec with three or two pulses than control (0 kV), and they decreased at weaker electric pulse conditions (Table S3, Fig. S3).

After adenovirus infection, on the other hand, we observed no GFP signal in cloudy catshark cells (Fig. S1d). Adenovirus is known to infect and induce gene expression in teleost cell lines and in zebrafish and medaka *in vivo* (Kawasaki et al., 2009; Overturf et al., 2003). This raises the possibility that infectivity of adenovirus is low in cartilaginous fish cells. Alternatively, the culture conditions with high osmolality, high urea, and low temperature might have reduced infectivity of adenovirus in the present study.

Meanwhile, PEI transfection, which is a method of chemical gene transfer similar to lipofection, failed to introduce an exogenous gene into cloudy catshark cells (Fig. S1c). In chemical transfection methods such as lipofectamine and PEI, it has been reported that transfected plasmids

were incorporated to nucleus at cell division (Haraguchi et al., 2022; Zabner et al., 1995). In this culture, it is possible that cell division did not occur sufficiently because the culture medium used did not contain trimethylamine-N-oxide (TMAO), or growth factors that are originally present in the cartilaginous fish body fluid. Future experiments may improve the efficiency of gene transfer by using previously reported culture conditions such as dogfish shark cell line and cloudy catshark lymphocytes (Parton et al., 2007; Uno et al., 2020).

REFERENCES

Haraguchi, T., Koujin, T., Shindo, T., Bilir, Ş., Osakada, H., Nishimura, K., . . . Hiraoka, Y. (2022). Transfected plasmid DNA is incorporated into the nucleus via nuclear envelope reformation at telophase. *Communications Biology, 5*(1), 78. doi:10.1038/s42003-022-03021-8

Kawasaki, T., Saito, K., Mitsui, K., Ikawa, M., Yamashita, M., Taniguchi, Y., . . . Sakai, N. (2009). Introduction of a Foreign Gene into Zebrafish and Medaka Cells Using Adenoviral Vectors. *Zebrafish, 6*(3), 253-258. doi:10.1089/zeb.2009.0596

Overturf, K., LaPatra, S., & Reynolds, P. N. (2003). The effectiveness of adenoviral vectors to deliver and express genes in rainbow trout, *Oncorhynchus mykiss* (Walbaum). *Journal of Fish Diseases, 26*(2), 91-101. doi:https://doi.org/10.1046/j.1365-2761.2003.00436.x

Parton, A., Forest, D., Kobayashi, H., Dowell, L., Bayne, C., & Barnes, D. (2007). Cell and molecular

biology of SAE, a cell line from the spiny dogfish shark, *Squalus acanthias*. *Comparative Biochemistry and Physiology Part C: Toxicology & Pharmacology, 145*(1), 111-119. doi:https://doi.org/10.1016/j.cbpc.2006.07.003

Toyoda, Y., Takada, T., Miyata, H., Ishikawa, T., & Suzuki, H. (2016). Regulation of the Axillary Osmidrosis-Associated ABCC11 Protein Stability by *N*-Linked Glycosylation: Effect of Glucose Condition. *PLoS One, 11*(6), e0157172. doi:10.1371/journal.pone.0157172

Uno, Y., Nozu, R., Kiyatake, I., Higashiguchi, N., Sodeyama, S., Murakumo, K., . . . Kuraku, S. (2020). Cell culture-based karyotyping of orectolobiform sharks for chromosome-scale genome analysis. *Communications Biology, 3*(1), 652. doi:10.1038/s42003-020-01373-7

Zabner, J., Fasbender, A. J., Moninger, T., Poellinger, K. A., & Welsh, M. J. (1995). Cellular and molecular barriers to gene transfer by a cationic lipid. *J Biol Chem, 270*(32), 18997-19007. doi:10.1074/jbc.270.32.18997
