# Supplementary material for: In vitro and in vivo gene transfer in the cloudy catshark Scyliorhinus torazame
Source: Dev Growth Differ. 2022 Nov 30;64(9):558–65. doi: 10.1111/dgd.12824 (PMC10099843; doi:10.1111/dgd.12824)
Supplement: Supplementary file 2 — Figure S1. Gene transfer into primary cultured cells of cloudy catshark by various methods. Figure S2. Efficiency of baculovirus‐mediated GFP transfection at various doses in vitro. Figure S3. Efficiency of gene transfer under various electroporation conditions in cloudy catshark cells in vitro. Figure S4. Results of electroporation under various conditions in cloudy catshark embryos in vivo. Figure S5. Spermatocyst morphology of the adult cloudy catshark testis. Table S1. The proportions of fluorescent cells after transfection of GFP‐containing vector or virus by various methods. Table S2. The proportions of fluorescent cells after infection with different volumes of baculovirus. Statistical significance of differences from the control (cells treated with 0 L baculovirus) was assessed by Dunnett's multiple comparison test (*p < .05, **p < .01). Table S3. The proportions of fluorescent cells after electroporation under various conditions. Statistical significance of differences from the control (cells subjected to electroporation at 0 kV) was assessed by Dunnett's multiple comparison test (*p < .05, **p < .01). [file DGD-64-558-s001.pdf]

Figure S1

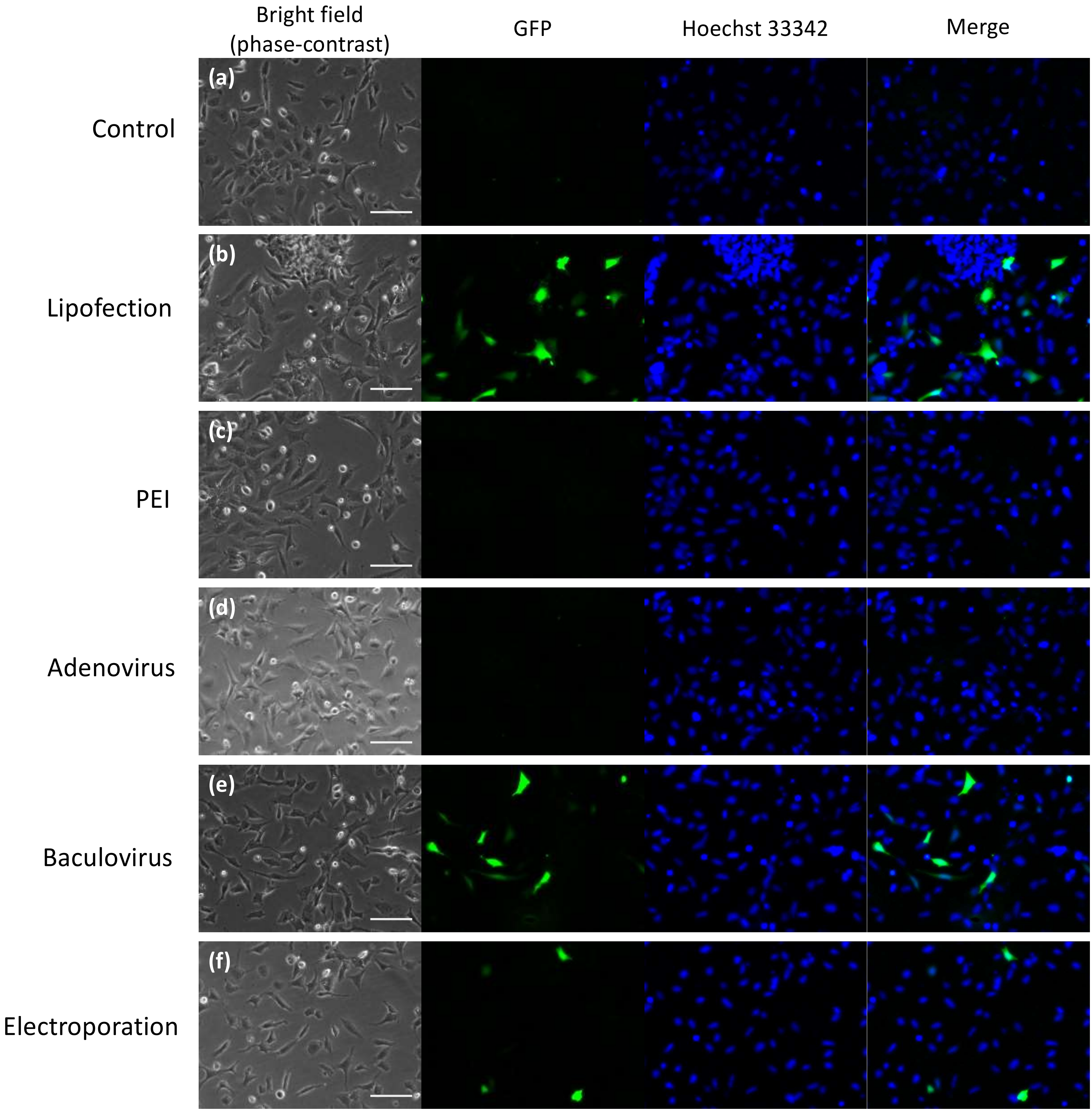
Bright field (phase-contrast)
GFP
Hoechst 33342
Merge
(a)
Control
(b)
Lipofection
(c)
PEI
(d)
Adenovirus
(e)
Baculovirus
(f)
Electroporation

Fig. S1. Gene transfer to primary cultured cells of cloudy catshark by various methods.

Left images of (a-f) shows the morphology of primary cultured cells after transfection, and center left images were their GFP fluorescence images. Their nuclei were counterstained with Hoechst 33342 (a-f, center right images) and the right columns show the merged images with GFP fluorescence (a-f). Primary cultured cells were transfected by lipofection (b), PEI (c), adenovirus (d), baculovirus (e) or electroporation (f). In the lipofection-treated group (b), the first fluorescent cells were observed two days after transfection. In baculovirus-infected cells (e), the first GFP-expressing cells appeared four days after infection. Electroporation-treated cells (f) also showed GFP signals from five days after electroporation. Note that these figures represent areas where GFP fluorescence was observed at relatively high frequency. (a) represents untransfected cells. Scale bars represent 100 μm.

Figure S2

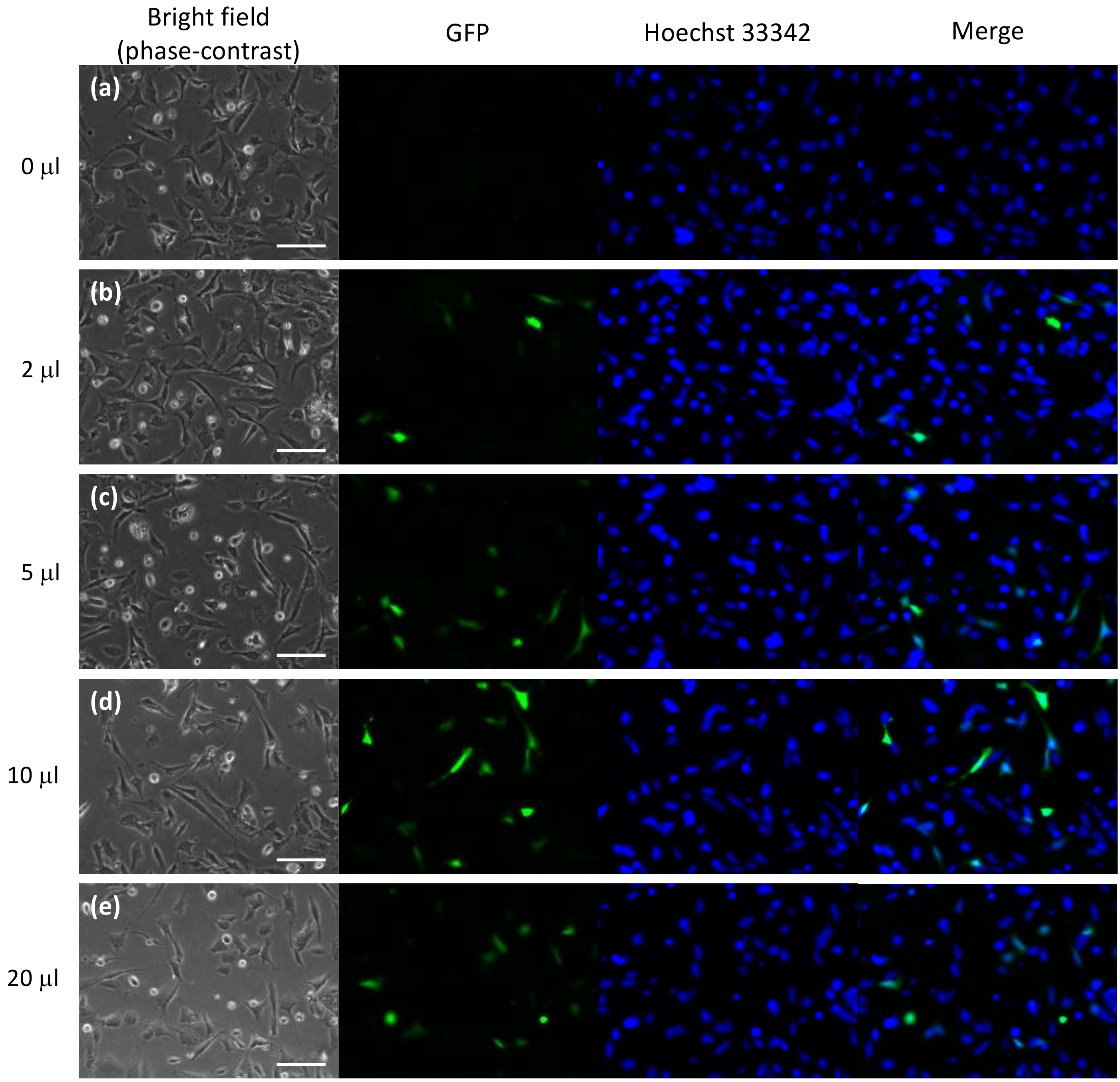
Bright field
(phase-contrast)
GFP
Hoechst 33342
Merge
(a)
(b)
(c)
(d)
(e)
0 μl
2 μl
5 μl
10 μl
20 μl

Fig. S2. Efficiency of baculovirus-mediated GFP transfection at various doses *in vitro*.

Phase-contrast images (a-e, left images) and the corresponding GFP fluorescent images (a-e, center left images) seven days after infection with various quantities of baculovirus. Hoechst 33342 was used for nuclear counterstaining (a-e, center right images) and merged images with GFP fluorescence were shown in most right images of (a-e). The volume of baculovirus solution: (a), 0 μl; (b), 2 μl; (c), 5 μl; (d), 10 μl; (e), 20 μl. Note that these figures represent areas where GFP fluorescence was observed at relatively high frequency. The titer of the P2 supernatant of baculovirus on primary cultured cells from cloudy catshark embryos and HEK293A cells was 1.36×10^5 gtu/ml and 1.55×10^5 gtu/ml, respectively. Scale bars represent 100 μm.

Figure S3

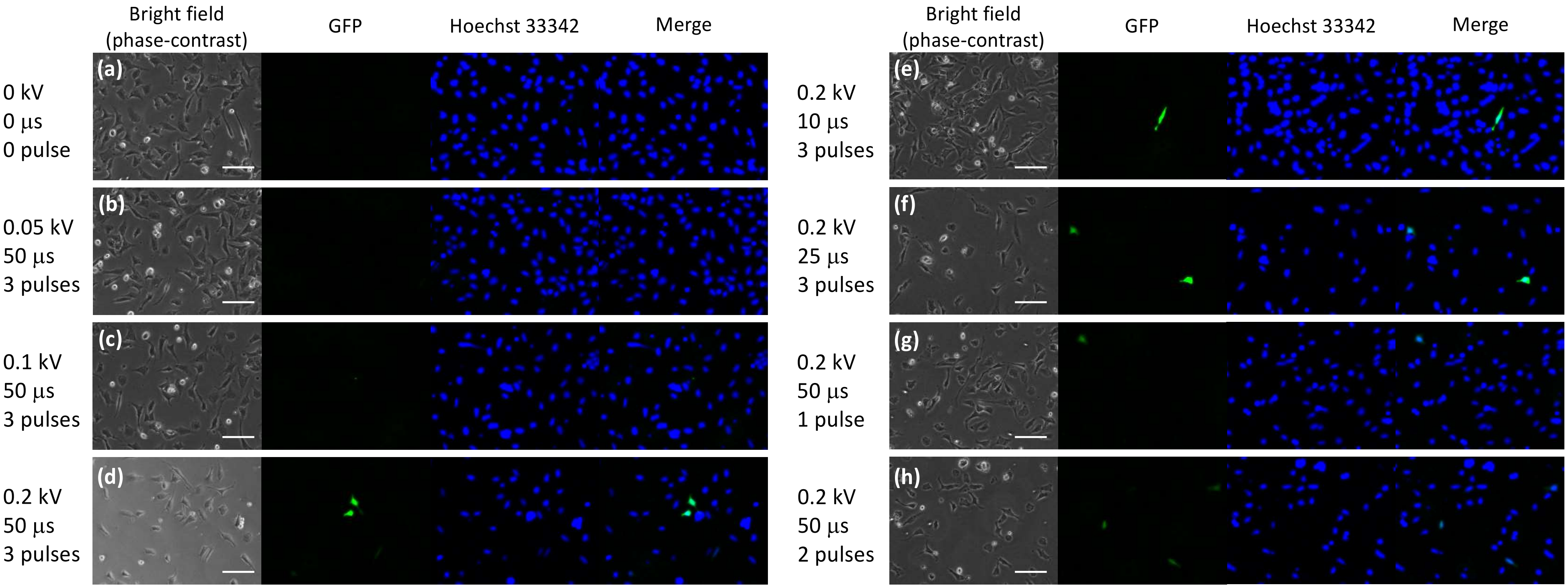

Bright field (phase-contrast)
GFP
Hoechst 33342
Merge
0 kV
0 μs
0 pulse
(a)
0.05 kV
50 μs
3 pulses
(b)
0.1 kV
50 μs
3 pulses
(c)
0.2 kV
50 μs
3 pulses
(d)
Bright field (phase-contrast)
GFP
Hoechst 33342
Merge
0.2 kV
10 μs
3 pulses
(e)
0.2 kV
25 μs
3 pulses
(f)
0.2 kV
50 μs
1 pulse
(g)
0.2 kV
50 μs
2 pulses
(h)

Fig. S3. Efficiency of gene transfer under various conditions of electroporation in cloudy catshark cells *in vitro*.

Phase-contrast images of cell morphology (a-h, left images) and the corresponding fluorescence images (a-h, center left images) nine days after electroporation. Nuclear counterstaining images using Hoechst 33342 and their merged images with GFP fluorescence were shown in second images from center right and right images of (a-h), respectively. Electroporation was performed using different voltages (a, 0 kV; b, 0.05 kV; c, 0.1 kV; d, 0.2 kV), durations (a, 0 μs; e, 10 μs; f, 25 μs; d; 50 μs) and pulse numbers (a, 0 pulse; g, 1 pulse; h, 2 pulses; d, 3 pulses). Note that these figures represent areas where GFP fluorescence was observed at relatively high frequency. When electroporation was performed at 0.2 kV, 50 μsec, 3 pulses (d) and 0.2 kV, 50 μsec, 2 pulses (h), the percentages of GFP-expressing cells were significantly higher than they were in the control (a). Scale bars represent 100 μm.

Figure S4

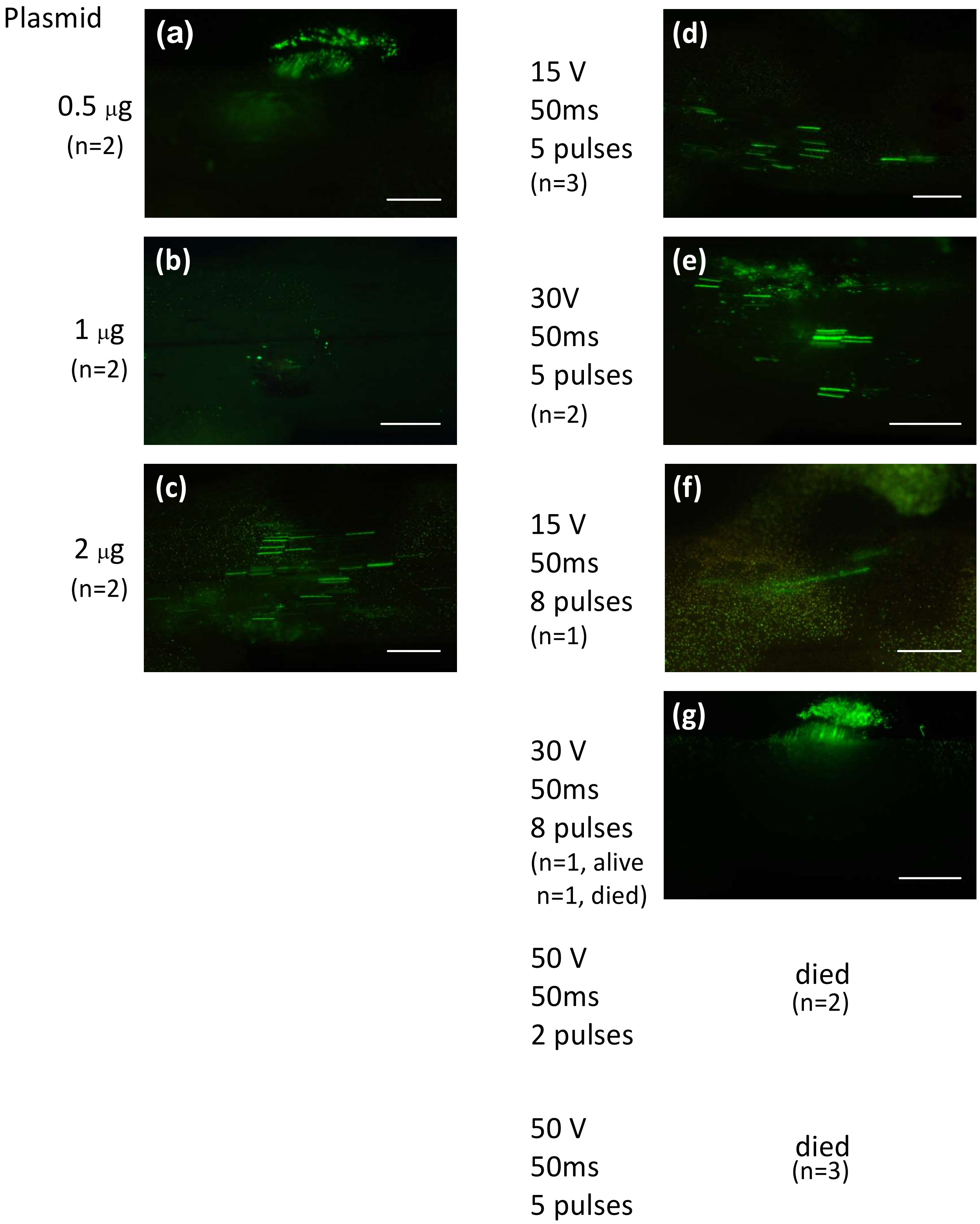
Plasmid
(a)
0.5 μg
(n=2)
(b)
1 μg
(n=2)
(c)
2 μg
(n=2)
(d)
15 V
50ms
5 pulses
(n=3)
(e)
30V
50ms
5 pulses
(n=2)
(f)
15 V
50ms
8 pulses
(n=1)
(g)
30 V
50ms
8 pulses
(n=1, alive
n=1, died)
50 V
50ms
2 pulses
died
(n=2)
50 V
50ms
5 pulses
died
(n=3)

Fig. S4. Results of electroporation with various conditions in cloudy catshark embryos *in vivo*.

Fluorescence images of cloudy catshark embryos seven days after electroporation. Electroporation was performed using different plasmid volume (a, d, e, f, g, 0.5 μg; b, 1 μg; c, 2 μg), voltage (d, f, 15 V; e, g, 30 V), and pulse numbers (d, e, 5 pulses; f, g, 8 pulses). Note that the electroporated embryos at 50 V, 50 ms, 2 pulses (two of two embryos), 50 V, 50 ms, 5 pulses (three of three embryos), 30 V, 50 ms, 8 pulses (one of two embryos) died within seven days of electroporation. Scale bars represent 1 mm.

Figure S5

(a)

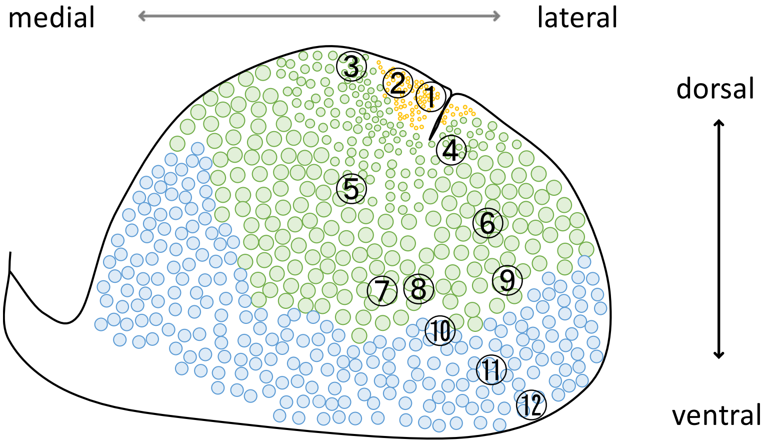

medial
lateral
dorsal
ventral
1
2
3
4
5
6
7
8
9
10
11
12

(b)

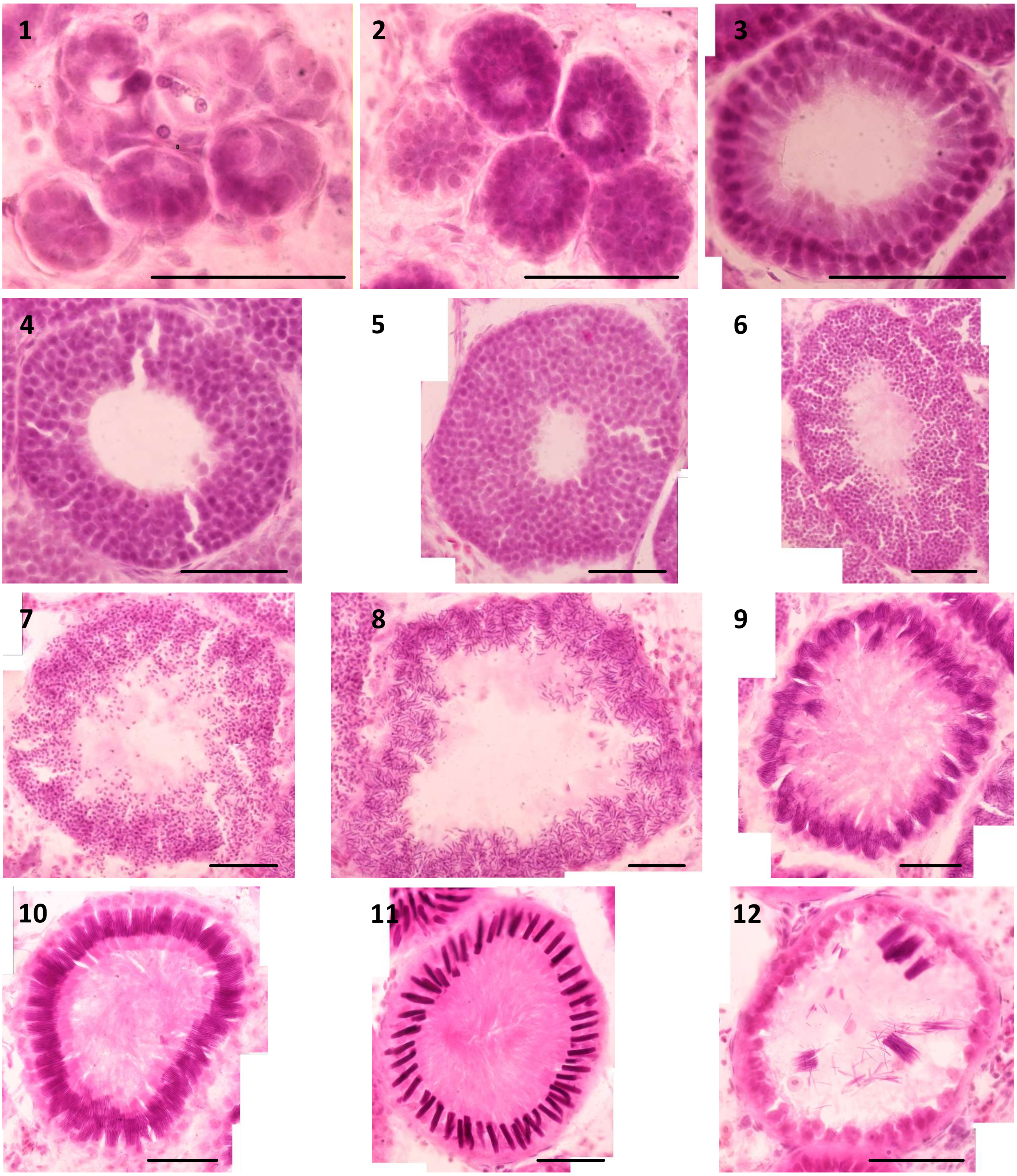

1
2
3
4
5
6
7
8
9
10
11
12

Fig. S5. Morphology of spermatocyst of adult cloudy catshark testis.

(a) Schematic diagram of transverse section of adult cloudy catshark testis. Spermatocysts containing more undifferentiated germ cells were localized in the dorsal side of the testis (yellow circles). The gradual distribution of the differentiation stage suggests that the spermatocysts migrate toward the radial side as the germ cells differentiate into meiosis (green circles) and spermiogenesis stage (blue circles). (b) Spermatocysts at different stages of spermatogenesis. The number in each panel represents the position in the testis and corresponds to the number in (a). Scale bars represent 100 μm.

Table S1. The proportions of cells with fluorescence after GFP-containing vector or virus transfection by various methods.

Primary cell cultures of cloudy catshark embryos contained very small numbers of cells showing autofluorescence. Since it was difficult to distinguish uncharacterized autofluorescence from GFP signals, we counted the total numbers of fluorescence-positive cells including both GFP-fluorescent and autofluorescent cells.

	(number of fluorescent cells/all cells)	% of fluorescent cells	n
Control	(0/5295)	0.01 ± 0.02	4
	(0/5866)		
	(2/5152)		
	(0/5443)		
Lipofection	(124/4688)	3.6 ± 1.3	4
	(185/3474)		
	(111/2934)		
	(84/3300)		
PEI †	(0/5377)	0.005 ± 0.009	4
	(0/6732)		
	(1/5519)		
	(0/4826)		
Adenovirus	(0/5474)	0 ± 0	4
	(0/6083)		
	(0/5511)		
	(0/5879)		
Baculovirus	(83/5075)	1.5 ± 0.48	4
	(121/5886)		
	(57/5902)		
	(68/5625)		
Electroporation	(50/4960)	0.59 ± 0.37	3

	(18/4328)		
	(18/5344)		

† polyethylenimine.

Table S2. The proportions of fluorescent-positive cells after infection with different volumes of baculovirus.

Statistically significant difference from control (cells treated with 0 μl baculovirus) was assessed by Dunnett's multiple comparison test (*$p < 0.05$, **$p < 0.01$).

Baculovirus (μl)	(number of fluorescent cells/all cells)	% of fluorescent cells	n
control (0 μl)	(1/6690)	0.008 ± 0.010	4
	(0/6968)		
	(0/5960)		
	(1/5421)		
2	(28/5910)	0.46 ± 0.15	4
	(38/6591)		
	(13/5208)		
	(35/6390)		
5	(58/6200)	1.22 ± 0.49	4
	(105/5880)		
	(37/5354)		
	(90/6223)		
10	(101/6239)	2.0 ± 1.1*	4
	(178/4935)		
	(83/5330)		
	(82/6026)		
20	(116/6058)	2.6 ± 1.5**	4
	(266/5599)		
	(55/3105)		
	(96/5310)		

Table S3. The proportions of cells with fluorescence after electroporation under various conditions. Statistically significant difference from control (cells subjected to 0 kV electroporation) was assessed by Dunnett's multiple comparison test (*p < 0.05, **p < 0.01).

Voltage (kV)	Length (μs)	Number	(number of fluorescent cells/all cells)	% of fluorescent cells	n
0	0	0	(5/8362)	0.02 ± 0.03	4
			(0/6224)		
			(0/6397)		
			(0/7587)		
0.05	50	3	(0/8858)	0 ± 0	4
			(0/5882)		
			(0/5774)		
			(0/7760)		
0.1	50	3	(2/9491)	0.04 ± 0.02	4
			(4/6516)		
			(3/5147)		
			(1/6780)		
0.2	50	3	(1/8468)	0.3 ± 0.2*	4
			(23/4507)		
			(12/4215)		
			(8/3499)		
0.2	10	3	(3/8261)	0.07 ± 0.07	4
			(11/6290)		
			(2/6207)		
			(2/7552)		
0.2	25	3	(7/6882)	0.2 ± 0.1	4
			(17/4547)		
			(7/3699)		
			(12/7213)		
0.2	50	1	(5/8196)	0.1 ± 0.05	4
			(11/5788)		
			(5/4383)		

			(9/6793)		
0.2	50	2	(13/6628)	0.3 ± 0.1**	4
			(21/4577)		
			(9/5846)		
			(14/5084)		
